# Depressive symptoms as a side effect of Interferon-α therapy induced by induction of indoleamine 2,3-dioxygenase 1

**DOI:** 10.1038/srep29920

**Published:** 2016-07-20

**Authors:** Yuki Murakami, Takaaki Ishibashi, Eiichi Tomita, Yukio Imamura, Tomoyuki Tashiro, Kanitta Watcharanurak, Makiya Nishikawa, Yuki Takahashi, Yoshinobu Takakura, Satoko Mitani, Hidetsugu Fujigaki, Yoshiji Ohta, Hisako Kubo, Takayoshi Mamiya, Toshitaka Nabeshima, Hyoung-Chun Kim, Yasuko Yamamoto, Kuniaki Saito

**Affiliations:** 1 Human Health Sciences, Graduate School of Medicine and Faculty of Medicine, Kyoto University, Kyoto 606-8507, Japan; 2 Organization for Research Initiatives and Development, Doshisha University, Kyoto 610-0394, Japan; 3 Department of Gastroenterology, Gifu Municipal Hospital, Gifu 500-8513, Japan; 4 Labratory of Nano-bio Probe, Quantitative Biology Center (QBic), RIKEN, Osaka 565-0874, Japan; 5 Department of Biopharmaceutics and Drug Metabolism, Graduate School of Pharmaceutical Sciences, Kyoto University, Kyoto 606-8501, Japan; 6 Department of Nursing School of Health Sciences, Gifu University of Medical Science, Gifu 501-3892, Japan; 7 Department of Medical Science and Technology, Faculty of Health Sciences, Hiroshima International University, Hiroshima, 739-2695, Japan; 8 Department of Medical Sciences Innovation, Fujita Health University Graduate School of Health Sciences, Aichi 470-1192, Japan; 9 Department of Chemical Pharmacology, Faculty of Pharmacy, Meijyo University, Aichi 468-8503, Japan; 10 Japanese Drug Organization of Appropriate Use and Research, Aichi 468-0069, Japan; 11 Advanced Diagnostic System Research Laboratory, Fujita Health University Graduate School of Health Science, Aichi 470-1192, Japan; 12 Aino University, Osaka 567-0012, Japan; 13 Neuropsychopharmacology & Toxicology Program, College of Pharmacy, Kangwon National University, Gangwon 200-701, Republic of Korea

## Abstract

Depression is known to occur frequently in chronic hepatitis C viral (HCV) patients receiving interferon (IFN)-α therapy. In this study, we investigated whether indoleamine 2,3-dioxygenase1 (IDO1)-mediated tryptophan (TRP) metabolism plays a critical role in depression occurring as a side effect of IFN-α therapy. Increases in serum kynurenine (KYN) and 3-hydroxykynurenine (3-HK) concentrations and in the ratios of KYN/TRP and 3-HK/kynurenic acid (KA) were much larger in depressive HCV patients than in non-depressed patients following therapy. Furthermore, transfection of a plasmid continuously expressing murine IFN-γ into normal mice significantly increased depression-like behavior. *IFN*-γ gene transfer also resulted in a decrease in serum TRP levels in the mice while KYN and 3-HK levels were significantly increased in both serum and frontal cortex. Genetic deletion of IDO1 in mice abrogated both the increase in depression-like behavior and the elevation in TRP metabolites’ levels, and the turnover of serotonin in the frontal cortex after *IFN*-γ gene transfer. These results indicate that the KYN pathway of IDO1-mediated TRP metabolism plays a critical role in depressive symptoms associated with IFN-α therapy.

Hepatitis C viral (HCV) infection affects approximately 170 million people worldwide^1^. Up to 85% of HCV-infected individuals may develop long-term chronic hepatitis C (CHC)^2^,^3^,^4^. Interferon-α (IFN-α) is a cytokine associated with early viral infection and has both anti-viral and anti-proliferative properties^5^. The current standard of treatment for CHC consists of combination therapy with IFN-α plus ribavirin, which has a broad spectrum anti-viral effect. In clinical trials, more than 50% of CHC patients treated with the combination therapy achieved a sustained viral response, defined as undetectable HCV in the blood 6 months after the end of the therapy^2^,^6^. Despite the potential therapeutic benefits of IFN-α, its administration often causes not only somatic symptoms, e.g., anorexia, insomnia, pain, and fever, but also neuropsychiatric symptoms, including depressive states, anhedonia, anxiety, and cognitive impairment^7^,^8^,^9^,^10^,^11^. Depression, a serious and frequently occurring side effect of IFN-α therapy, is one of the major reasons for cessation of the therapy^10^,^12^,^13^,^14^,^15^. In order to avoid the discontinuation of IFN-α therapy due to its depressive side effects, it is important to identify the contributing factor(s) leading to the depressive symptoms.

The metabolism of L-tryptophan (TRP), an essential amino acid, in extrahepatic tissues proceeds through the L-kynurenine (KYN) and the serotonin [5-hydroxytryptamine (5-HT)] pathways (see Supplemental Fig. 1S). Indoleamine 2,3-dioxygenase1 (IDO1) is the first and rate-limiting enzyme in the KYN pathway and is induced by several pro-inflammatory cytokines, including IFNs (IFN-α, β, γ), tumor necrosis factor (TNF-α), and interleukin 6 (IL-6) (see Supplemental Fig. 1S)^16^,^17^. Recent preclinical studies in mice have demonstrated that either pharmacological inhibition of IDO1 enzymatic activity or genetic deletion of *IDO1* abrogates acute and chronic inflammation-dependent behavioral changes induced by peripheral or central administration of lipopolysaccharide (LPS)^18^,^19^,^20^,^21^,^22^. Direct activation of the central cytokine signaling pathway by intracerebroventricular administration of TNF-α, LPS, or the human immunodeficiency virus transactivator of transcription in mice develops depression-like behavior through the up-regulation of IDO1^22^,^23^,^24^,^25^. Additionally, it has been reported that peripheral administration of KYN alone can induce depression-like behavior in rats^26^. KYN in blood is taken up into the brain through the blood-brain barrier and is a primary source of several metabolites of the KYN pathway in the central nervous system (CNS)^27^,^28^. In a clinical study, patients with IFN-α therapy show increases in the total Montgomery-Asberg Depression Rating Score (MADRS), an index of depressive symptoms, as well as in the KYN/TRP ratio (reflecting IDO1 activity) and the KYN/kynurenic acid (KA) ratio (reflecting the neurotoxic challenge)^29^, although the TRP/competing amino acid (CAA) ratio (reflecting TRP availability to the brain) does not significantly change during therapy. These findings suggested that TRP depletion itself may not be required for the induction of behavioral changes due to IDO1 activation and that KYN and its neuroactive metabolites are more relevant than TRP depletion to cytokine-induced behavioral changes. However, it is still unclear whether direct induction of IDO1 and TRP metabolites plays a critical role in depressive symptoms induced by IFN-α therapy.

Major depression is accompanied by dysfunction of the serotonergic neuronal system. This includes, for example, decreased activity of the central presynaptic 5-HT neurons, which is related to a decreased availability of plasma TRP, and changes in postsynaptic receptors^30^. Further, treatment of rats with IFN-α significantly reduces 5-HT in the frontal cortex in a dose-dependent manner^31^. A clinical study also shows immunotherapy with IFN-α significantly increases the severity of depressive symptoms related to the depletion of serum 5-HT and induction of the catabolism of TRP to KYN^15^. These studies suggested that IFN-α-induced changes in the serotonergic neuronal function could play a role in the development of IFN-α-induced depressive symptoms. However, whether the induction of IDO1 and serotonergic turnover induced by immune activation are directly related is still unclear.

In the present study, we examined changes in the serum levels of TRP and its metabolites in patients with HCV before and during IFN-α therapy. We also investigated the relationship between changes in serum KYN/TRP and 3-HK/KA ratios to incidence of depression in patients receiving IFN-α therapy. Mouse IDO1 is induced by IFN-γ more markedly than IFN-α^32^. Although IFN-α has a weak direct IDO1-stimulating effect, it enhances IDO1 activity indirectly by stimulating IFN-γ production^33^,^34^. Therefore, we investigated whether IDO1 activity induced by *IFN*-γ gene transfer impaired behavior in mice. Metabolic abnormalities of the KYN pathway have been detected in the frontal cortex in a murine model of major depression^35^. Thus, we also measured the concentration of TRP metabolites in the serum and frontal cortex of normal mice after *IFN*-γ gene transfer. Finally, we examined the effect of *IDO1* gene-deficiency on depression-like behavior, the levels of TRP metabolites, and changes of 5-HT turnover in the frontal cortex after *IFN*-γ gene transfer.

## Results

### Clinical characteristics of HCV patients undergoing IFN-α therapy

The characteristics of HCV patients who received IFN-α therapy are shown in Table 1. Among 49 HCV patients, 32 were male and 17 female. No significant differences were found between depression-negative (−) and depression-positive (+) HCV patients in their age, the ratio of gender, HCV genotype, or aspartate aminotransferase (AST) and alanine aminotransferase (ALT) levels (Table 1).

### Changes in the levels of serum TRP and its metabolites in HCV patients receiving IFN-α therapy

**Figure 1:**
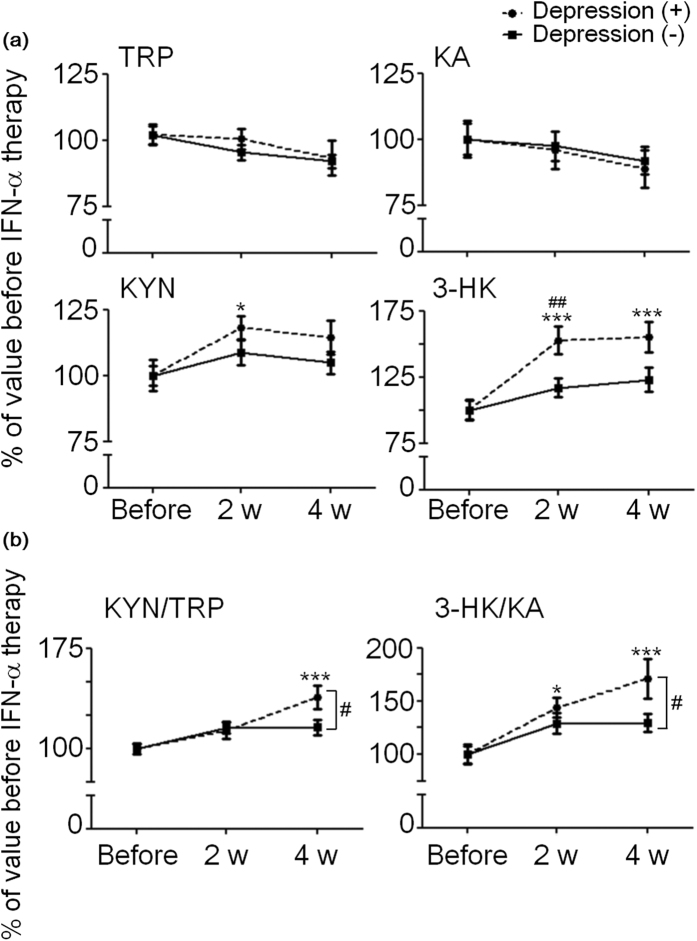
Changes in the levels of serum TRP and its metabolites in HCV patients undergoing IFN-α therapy. (**a**) The Y axis shows TRP, KYN, KA, and 3-HK concentrations in HCV patients at 2 and 4 weeks after the onset of therapy, expressed as a percentage of the concentration before IFN-α therapy. (**b**) Serum KYN/TRP and 3-HK/KA ratios in HCV patients are shown as a percentage of values before IFN-α therapy. Rectangles indicate non-depressive HCV patients [Depression (−)]; circles indicate HCV patients with depressive symptoms [Depression (+)]. Each data point represents the mean ± SEM of values obtained from n = 30 depression (−) patients and n = 19 depression (+) patients. ^*^p < 0.05, ^***^p < 0.001 *versus* before the onset of IFN-α therapy, ^#^*p* < 0.05, ^##^*p* < 0.01 *versus* depression (−) patients. Detailed statistical analyses are in Tables 2 and 3.

Figure 1a shows serum TRP, KYN, KA, and 3-HK concentrations in HCV patients before the onset of IFN-α therapy and at 2 and 4 weeks after the onset of therapy. In depression (+) patients, compared to values before the onset of therapy, serum TRP concentration tended to decrease while serum KYN concentration increased significantly at 2 weeks. Serum 3-HK concentration increased significantly at both 2 and 4 weeks after the onset of therapy. There was a no significant change in serum KA level, but it tended to decrease at either time point. In depression (−) patients, serum KYN and 3-HK concentrations increased at 2 and 4 weeks compared to values before the onset of therapy, but not significant. Further, serum TRP and KA concentrations tended to decrease, but not significant. During IFN-α therapy, the increased levels of serum KYN and 3-HK tended to be higher in depression (+) patients than in depression (−) patients and there were significant increases of 3-HK level in depression (+) patients than in depression (−) patients at 2 weeks after the onset of therapy (Fig. 1 and Table 2). No changes in serum anthranilic acid (AA) and 3-hydroxyanthranilic acid (3-HAA) levels were detected at 2 and 4 weeks during IFN-α therapy in both depression (+) and depression (−) patients (Table 2).

To determine the relationships between the changes in serum levels of TRP and its metabolites and the development of depression in HCV patients undergoing IFN-α therapy, serum KYN/TRP (reflecting IDO1 activity) and 3-HK/KA (reflecting neurotoxic indices) were examined. We investigated these ratios before the onset of the therapy and at 2 and 4 weeks after the onset of therapy. In depression (−) patients, serum KYN/TRP and 3-HK/KA ratios increased at 2 and 4 weeks after the onset of therapy, and the increased levels of these ratios were similar at both time points (Fig. 1b and Table 2b). In depression (+) patients, serum KYN/TRP ratio increased significantly at 4 weeks after the onset of therapy while the serum 3-HK/KA ratio increased significantly at both 2 and 4 weeks after the onset of therapy. Moreover, the increased levels of KYN/TRP and 3-HK/KA ratios tended to be higher in depression (+) patients than in depression (−) patients and there were significant increases of both ratios in depression (+) patients than in depression (−) patients at 4 weeks after the onset of therapy (Fig. 1b and Table 3).

### Abnormal behavior in a forced swimming test after *IFN*-γ gene transfer in mice

**Figure 2:**
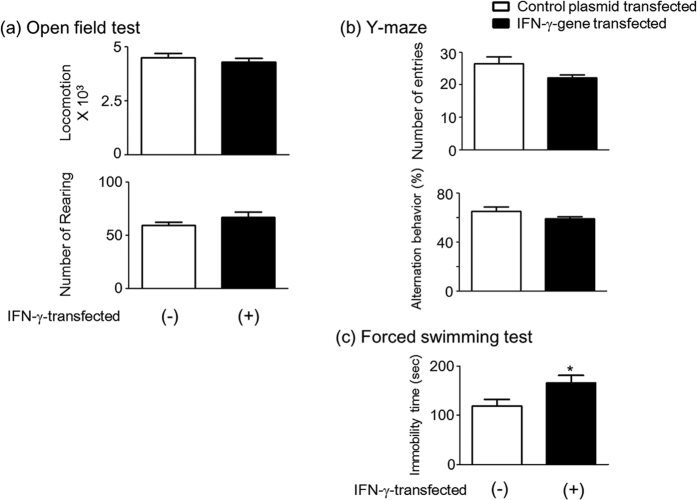
Abnormal behavior in a forced swimming test after *IFN*-γ gene transfer in mice. The open bar shows control plasmid (empty pCpG-mcs vector)-injected mice [IFN-γ transfected (−) mice]; closed bar shows mice received the pCpG-Muγ plasmid, which continuously expresses murine IFN-γ [IFN-γ-transfected (+) mice]. (**a**) Locomotor activity and rearing of IFN-γ-transfected (+) and (−) mice in a novel environment in an open field test. (**b**) Short-term memory in a Y-maze test for the two groups of mice. Alternation behavior and total arm entries were measured in an 8 min session. (**c**) Immobility of IFN-γ-transfected (+) and (−) mice in a forced swimming test. Each column represents the mean ± SEM (n = 9–16). *p < 0.05 *versus* IFN-γ-transfected (−) mice.

In order to clarify whether the induction of IDO1 by IFN-γ affects behavior, three tests, open-field test (OFT), the Y-maze test, and forced swimming test (FST), were conducted in mice. Mice were transfected with either a pCpG-Muγ plasmid that continuously expressed *IFN*-γ [IFN-γ transfected (+) mice] or a control plasmid (pCpG-mcs) that did not contain the *IFN*-γ gene [IFN-γ transfected (−) mice]. In the OFT, no significant differences in locomotion or the number of rearing were detected between IFN-γ transfected (+) and (−) mice (Fig. 2a). Similarly, in the Y-maze test, no significant differences in the number of arm entries and alternation behavior were found between the two groups of mice (Fig. 2b). However, in the FST, immobility time was significantly longer in IFN-γ transfected (+) mice (Fig. 2c, Student’s *t* test, *t*_(34)_ = 2.286, *p* = 0.0286).

### Changes in the levels of TRP and its metabolites in the serum and frontal cortex of mice after *IFN*-γ gene transfer

**Figure 3:**
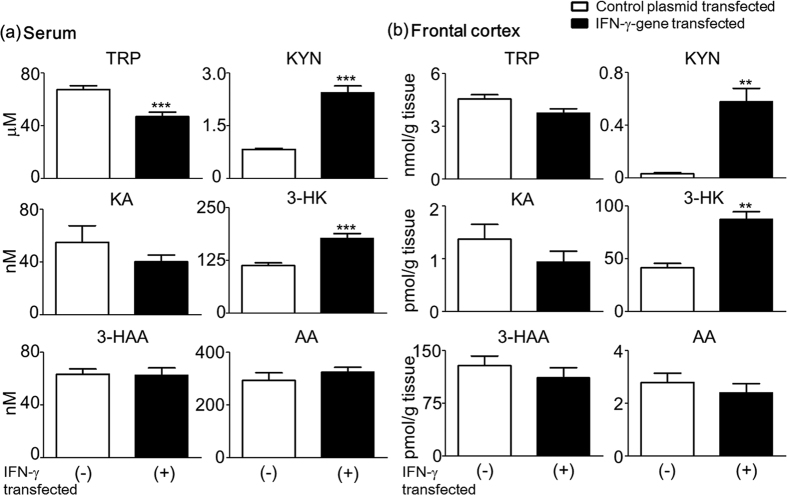
Changes in the levels of TRP and its metabolites in the serum and frontal cortex of mice after *IFN*-γ gene transfer. TRP-KYN metabolite concentrations were determined in the serum (**a**) and the frontal cortex (**b**) of mice at 28 days after *IFN*-γ-gene transfer. The open bar shows IFN-γ-transfected (−) mice, and the closed bar shows IFN-γ-transfected (+) mice. Each column represents the mean ± SEM (n = 15–20). **p < 0.01, ***p < 0.001 *versus* IFN-γ-transfected (−) mice.

Mice were euthanized immediately following behavioral testing for the determination of the levels of TRP, KYN, KA, 3-HK, 3-HAA, and AA in the serum and the frontal cortex. Serum TRP concentration was significantly lower in IFN-γ transfected (+) mice compared to IFN-γ transfected (−) mice. In contrast, KYN and 3-HK serum levels were significantly higher in the IFN-γ transfected (+) mice. However, there were no significant differences in KA, 3-HAA, and AA serum concentrations between the two groups (Fig. 3a). In the frontal cortex, IFN-γ transfected (+) mice had significantly higher KYN and 3-HK levels than the IFN-γ transfected (−) mice. There were no significant differences in TRP, KA, 3-HAA, and AA levels between the two groups, although the TRP and KA levels in the frontal cortex tended to be lower in the IFN-γ transfected (+) mice (Fig. 3b).

### Effects of *IDO1* gene-deficiency on depressive behavior, changes in TRP metabolism, serotonin levels and serotonin turnover in the mouse frontal cortex after *IFN*-γ-gene transfer

**Figure 4:**
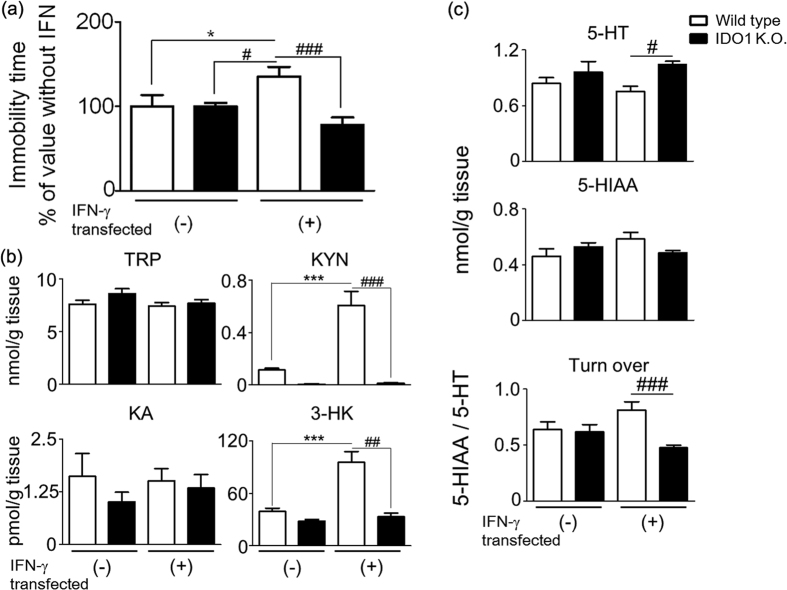
Effects of *IDO1* gene-deficiency on depressive behavior, changes in TRP metabolism, serotonin levels and serotonin turnover in the mouse frontal cortex after *IFN*-γ-gene transfer. (**a**) A forced swim test was performed 28 days after *IFN*-γ-gene transfer in wild type mice and *IDO1* K.O. mice. The Y axis shows % value of immobility time in IFN-γ-transfected (+) mice, compared with the time (100%) in IFN-γ-transfected (−) mice. Actual immobility times in IFN-γ-transfected (−) and (+)/wild type mice were 118.9 ± 162.1 and 160.9 ± 137.0 sec, respectively. While, actual immobility times in IFN-γ-transfected (−) and (+)/*IDO1* K.O. mice were 188.1 ± 78.0 and 146.5 ± 171.0 sec, respectively (n = 8–15). The level of TRP metabolites (**b**) and the amount of 5-HT, 5-HIAA, and 5-HIAA/5-HT ratio as an index of serotonin turnover (**c**) in the frontal cortex of mice at 28 days after *IFN*-γ-gene transfer (n = 6–15). The open bar shows wild type; the closed bar, *IDO1* K.O. mice. IFN-γ-transfected (−) mice were injected with the control plasmid (pCpG-mcs), and IFN-γ-transfected (+) mice were injected with the IFN-γ-expressing pCpG-Muγ plasmid. Each column represents the mean ± SEM. *p < 0.05, ***p < 0.001 *versus* IFN-γ-transfected (−) wild type mice, ^#^*p* < 0.05, ^##^*p* < 0.01, ^###^*p* < 0.001 *versus* IFN-γ-transfected (+) wild type mice.

We evaluated the role of IDO1 in the development of depressive-like behavior after *IFN*-γ gene transfer using *IDO1* gene knockout (K.O.) mice. Mice were subjected to a forced swimming test as described above. The increase of time spent in an immobile posture in the *IFN*-γ transfected (+)/wild type mice was significantly improved in *IDO1* K.O. mice (Fig. 4a, two-way ANOVA, F_*IDO1* KO (1, 33)_ = 10.84, *p* = 0.0024; F_IFN-_γ _(1, 33)_ = 0.16, *p* = 0.6962, F_*IDO1* KO x IFN-_γ _(1, 33)_ = 7.88, *p* = 0.0083).

In wild type mice, *IFN*-γ gene transfer significantly increased the concentrations of KYN and 3-HK in the frontal cortex by a factor of 4.7- and 2.5-fold, respectively, but had no significant effect on TRP and KA (Fig. 4b). In contrast, *IDO1* K.O. mice reversed these changes in *IFN*-γ gene transfer mice (Fig. 4b). After *IFN*-γ gene transfer, KYN and 3-HK levels in the frontal cortex were significantly lower in *IDO1* K.O. mice than in wild type mice (for KYN, two-way ANOVA, F_*IDO1* KO (1, 36)_ = 57.67, *p* < 0.0001; F_IFN-_γ _(1, 36)_ = 28.70, *p* < 0.0001, F_*IDO1* KO x IFN-_γ _(1, 36)_ = 27.20, *p* < 0.0001; for 3-HK, two-way ANOVA, F_*IDO1* KO (1, 14)_ = 44.52, *p* < 0.0001; F_IFN-_γ _(1, 14)_ = 31.15, *p* < 0.0001, F_*IDO1* KO x IFN-_γ _(1, 14)_ = 21.66, *p* = 0.0004), but there were no significant differences in TRP and KA levels between the two groups (for TRP, two-way ANOVA, F_*IDO1* KO (1, 23)_ = 1.86, *p* = 0.1855; F_IFN-_γ _(1, 23)_ = 1.51, *p* = 0.2314, F _*IDO1* KO x IFN-_γ _(1, 23)_ = 0.65, *p* = 0.4291; for KA, two-way ANOVA, F_*IDO1* KO (1, 19)_ = 0.72, *p* = 0.4052; F_IFN-_γ _(1, 19)_ = 0.06, *p* = 0.812, F_*IDO1* KO x IFN-_γ _(1, 19)_ = 0.22, *p* = 0.6432).

To study the neurochemical basis of depression-like behavior in wild type mice after *IFN*-γ gene transfer, the amounts of 5-HT and its metabolite 5-hydroxyindoleacetic acid (5-HIAA) in the frontal cortex of wild type and *IDO1* K.O. mice were compared (Fig. 4c). Compared to IFN-γ transfected (−)/wild type mice, the level of 5-HT was slightly decreased and the level of 5-HIAA was increased in the frontal cortex of IFN-γ transfected (+)/wild type mice. Therefore, the 5-HIAA/5-HT ratio increased, reflecting the turnover of 5-HT in IFN-γ transfected (+)/wild type mice (Fig. 4c). In the frontal cortex of IFN-γ transfected (−)/*IDO1* K.O. mice, 5-HT and 5-HIAA levels were slightly higher compared to IFN-γ transfected (−)/wild type mice with no significant change in 5-HIAA/5-HT ratio. After *IFN*-γ gene transfer, 5-HT levels in the frontal cortex of IFN-γ transfected (+)/*IDO1* K.O. mice were significant higher, although there was a slight decrease in 5-HIAA content compared with that of IFN-γ transfected (+)/wild type mice (Fig. 4c, for 5-HT, two-way ANOVA, F_*IDO1* KO (1, 49)_ = 8.73, *p* = 0.0048; F_IFN-_γ _(1,49)_ = 0.00, *p* = 0.9643, F_*IDO1* KO x IFN-_γ _(1, 49)_ = 1.39, *p* = 0.2448; for 5-HIAA, two-way ANOVA, F_*IDO1* KO (1, 50)_ = 0.17, *p* = 0.6852; F_IFN-_γ _(1,50)_ = 1.05, *p* = 0.3098, F_*IDO1* KO x IFN-_γ _(1, 50)_ = 4.57, *p* = 0.0374). 5-HT turnover (i.e., the 5-HIAA/5-HT ratio) was significantly lower in the frontal cortex of IFN-γ transfected (+)/*IDO1* K.O. mice compared to that of IFN-γ transfected (+)/wild type mice (Fig. 4c, two-way ANOVA, F_*IDO1* KO (1, 49)_ = 8.71, *p* = 0.0048, F_IFN-_γ _(1,49)_ = 0.08, *p* = 0.7749, F_*IDO1* KO x IFN-_γ _(1, 49)_ = 6.94, *p* = 0.0112).

## Discussion

Previous studies suggested that IDO1-mediated TRP metabolism may be implicated in the development of depression, a side effect of IFN-α therapy in HCV patients. To further clarify the relationship between the IDO1-induced KYN pathway and the development of depressive symptoms during IFN-α therapy, we measured TRP metabolites of the KYN pathway in the serum of HCV patients undergoing IFN-α therapy. We found that HCV patients showed decreased TRP and increased KYN concentrations without any changes in KA, AA, and 3-HAA concentrations during IFN-α therapy (Fig. 1a and Table 2). Further, depression (+) patients showed a larger increase in 3-HK concentration compared to depression (−) patients during therapy (Table 2). Ogawa *et al*. recently showed that mean plasma TRP was significantly decreased in major depressive disorder (MDD) patients *versus* healthy controls^36^. Teraishi *et al*. also demonstrated increased TRP metabolism along the KYN–NAD pathway, but not the KYN–KA pathway, in patients with MDD^37^. Our results are consistent with these findings. We also compared the ratios of KYN/TRP (reflecting IDO1 activity) and 3-HK/KA (reflecting neurotoxic indices)^38^,^39^ between depression (+) and depression (−) patients during IFN-α therapy (Fig. 1b and Table 3). In both groups, the ratios of KYN/TRP and 3-HK/KA increased during therapy. However, in depression (+) patients, the ratios of KYN/TRP and 3-HK/KA increased by a much greater degree than in depression (−) patients during therapy (Table 3). In these patients, the serum KYN/TRP and 3-HK/KA ratios tended to further increase at the diagnosis of depression, but at 70.3 ± 9.1 days after the end of therapy, they returned to the same levels as before the onset of the therapy. The severity of depressive symptoms was not assessed during therapy, e.g., using neither the Hamilton Depressing Rating Scale nor MADRS, therefore, we could not clearly show the relationship between the exacerbation of depression and changes of TRP metabolites. However, our results suggest that HCV patients who have a high sensitivity for IDO1 induction by IFNs are more susceptible to the depression-related side effects of IFN-α therapy.

We also examined behavioral changes and TRP metabolites in IFN-γ-transfected (+) mice, which showed high induction of IDO1 in the brain. Previous studies have shown that all three IFNs (IFN-α, -β and -γ) induced strong IDO1 activity in human peripheral blood mononuclear cells^40^,^41^. In contrast, mouse IDO1 is induced more markedly by IFN-γ than IFN-α, which has only a weak direct IDO1-stimulating effect^32^. To confirm this, we administered a single i.p. injection of either mouse recombinant (mr) IFN-α or mrIFN-γ to mice to compare the induction of IDO1 activity (see Supplemental Fig. 3S). We found that mrIFN-γ significantly increased the activity of IDO1, whereas mrIFN-α showed a very weak IDO1 induction in mice, consistent with former research (see Supplemental Figs 3S and 4S)^32^. Additionally, our previous studies also demonstrated that the response of IDO1 activity to systemic and/or CNS immune activation and inflammation depends on species and cell types^17^. These results suggest that human cells, more so than mice cells, are highly responsive to IDO1 induction by IFN-α. We hypothesized that the high induction of IDO1 and the imbalance of TRP metabolites induced by IFNs in humans may be related to psychiatric side effects such as depression. Therefore, we selected *IFN*-γ gene transfer to induce a high IDO1 activity and cause the imbalance of TRP-KYN pathway rather than IFN-α in our animal model. However, species difference for the response of IDO1 induction might be one of the limitations in this study.

The current results indicate that IDO1 induction by IFN-γ is a critical factor in depressive-like behaviors but not in short-term memory or locomotor activity in mice. The induction of IDO1 by *IFN*-γ gene transfer significantly changed the levels of TRP and its metabolites in the serum and frontal cortex of mice (Fig. 3). These results suggest that an alternative interpretation for the involvement of IDO1 in IFN-γ-induced depressive-like behavior is the generation of neuroactive TRP metabolites. Although we cannot exclude the possibility that genetic deletion of *IDO1* and the resulting alterations in TRP metabolites affect other behavioral tests, our results show mice deficient in IDO1 do not develop depressive-like behavior and do not increase TRP metabolites after *IFN*-γ gene transfer (Fig. 4). This interpretation is consistent with our clinical data and previous studies by O’Connor *et al*. and Wichers *et al*.^18^,^29^.

An alternative interpretation for the involvement of IDO1 in cytokine-induced depression is the generation of neuroactive TRP metabolites. While KYN itself is not neuroactive, it readily crosses the blood brain barrier *via* the large neutral amino acid transporters^27^. In the brain, KYN is taken up by glia cells, where it is further metabolized into neuroactive compounds such as 3-HK, KA, which are direct metabolites of KYN, and quinolinic acids (QUIN). When IDO1 is upregulated, KYN metabolism shifts from the predominant production of KA toward the generation of increased amounts of 3-HK and QUIN. 3-HK and QUIN generate free radicals, and QUIN also acts as an N-methyl-D-aspartate (NMDA) receptor agonist. Both metabolites are considered excitotoxic compounds in the brain. Preclinical, *in vitro*, and post-mortem data demonstrate that the elevated concentrations of these neurotoxic KYN metabolites are associated with neuronal damage and/or suppression of neurogenesis. KA is a glutamate receptor antagonist, which is also reported to inhibit the alpha 7 nicotinic acetylcholine receptors. Earlier studies of KA demonstrated that it can be neuroprotective against neuronal damage caused by neurotoxic QUIN, an effect that is most likely mediated through inhibitory activity at NMDA receptors^42^. More recent studies have demonstrated decreases in plasma KA and the KA/QUIN ratio in patients with depression^39^,^43^ while increased KA levels have been reported in the cerebrospinal fluid and postmortem brain of schizophrenia patients. Sustaining physiological levels of KA are likely important in maintaining a basal neuroprotective environment within the brain, but pathophysiologically elevated levels of KA could contribute to cognitive deficits and a reduction of dopaminergic and glutamatergic neurotransmission^44^. Conceivably, the relative effects of 3-HK and QUIN *versus* KA are dependent on the type of disorder (e.g., MDD *versus* schizophrenia). Therefore, IFN-α treatment may directly and/or indirectly stimulate IDO1 induction and thereby promote the KYN pathway, which results in increased levels of neuroactive compounds and an imbalance of TRP metabolites in the brain. Neuroactive TRP metabolites such as 3-HK, QUIN, and KA may promote oxidative stress, cell death, and excessive glutamate release that can cause neuronal damage and behavioral changes^45^,^46^. More experiments blocking KYN metabolizing enzymes, e.g., kynurenine-3-monooxygenase (KMO) or kynurenine aminotransferase II (KAT II), will clarify whether KYN induces depressive-like behavior by itself or after further generation of its downstream neuroactive metabolites.

Other studies have emphasized that the serotonin (5-HT) pathway is also relevant to depression. Dysfunction of the 5-HT pathway contributes to the development of depressive symptoms, but many antidepressants affecting 5-HT reuptake attenuates the symptoms. Moreover, selective serotonin reuptake inhibitors (SSRIs) have been shown to improve depressive symptoms during IFN-α therapy^47^. As the level of 5-HT in peripheral blood may not reflect the actual level of 5-HT in the brain, we did not measure 5-HT level in the serum of HCV patients. However, TRP and 5-hydroxytryptophan, a precursor of 5-HT, were significantly decreased from baseline in the serum of HCV patients during IFN-α therapy^48^. Barton *et al*. demonstrated that brain 5-HT turnover was elevated in non-medicated patients with MDD and was influenced by the serotonin transporter (5-HTT) genotype^49^. Thus, we speculate that biological mechanisms underlying the IFN-α treatment induced-depressive symptoms are linked not only to the activated IDO1 and KYN pathway but also to a dysfunction of the 5-HT system.

IFN-γ-induced depressive-like behavior appears to be dependent on IFN-γ-induced changes in the KYN pathway *via* IDO1 induction and also on increased 5-HT turnover, a parameter of serotonergic neuronal activity (Fig. 4c). Increases in IDO1 activity have the potential to inhibit serotoninergic neurotransmission by decreasing the bioavailability of TRP. Although a significant decrease in peripheral levels of TRP was observed after *IFN*-γ gene transfer, brain TRP and 5-HT levels were not significantly affected. IFN-α and –γ are known to increase 5-HT reuptake and the transcription rate of 5-HTT mRNA^50^. The increase in 5-HT reuptake would be expected to decrease synaptic 5-HT. 5-HIAA is produced by intraneuronal deamination (*via* monoamine oxidase A) of 5-HT after either leakage of the neurotransmitter into the axoplasm from storage vesicles or reuptake of the neurotransmitter after exocytotic release. The contribution of the latter process is reflected by the decrease in 5-HIAA production after neuronal uptake blockade. We found that *IFN*-γ gene transfer caused a non-significant trend towards increased 5-HIAA levels in wild type mice but not in *IDO1* K.O. mice, indicative of a potential increase in IDO1-induced 5-HT turnover following *IFN*-γ gene transfer. An elevated 5-HT turnover suggests a process by which the availability of 5-HT to be released by neurons is lowered to compensate for neuronal dysfunction related to depressive-like behavior induced by *IFN*-γ gene transfer. Previous studies also have shown that brain 5-HT turnover is substantially elevated in non-medicated MDD patients and reduced following SSRI therapy^49^,^51^. Taken together, an alternative interpretation for the involvement of IDO1 in IFN-γ-induced depressive-like behavior may be that depression is related to not only the generation of neuroactive TRP metabolites but also the alteration of serotoninergic neurotransmission.

In conclusion, the present study indicates that IDO1 is a critical molecular regulator of IFNs-induced depressive symptoms. Moreover, the depressive symptoms are induced *via* increases in the degradation of TRP along the KYN pathway and changes in 5-HT turnover. Our findings suggest that inflammatory pathways that lead to the activation of IDO1 may be novel therapeutic targets in patients suffering from inflammation-associated depression, e.g., as is the case during HCV or cancer therapy. However, further insight into the role of each downstream KYN pathway metabolite in the pathological process is still needed to clarify the relationship of this pathway to various other neurotransmitters.

## Materials and Methods

### Samples

#### HCV Patients undergoing IFN-α therapy

Forty nine patients (32 males and 17 females; mean age 54.0 ± 2.3 years) suffering from chronic hepatitis C were recruited from Gifu Municipal Hospital, Gifu, Japan. Table 1 shows the clinical characteristics of patients with HCV. All patients received IFN-α therapy. In this study, 21 (42.9%) patients received recombinant (r) IFN-α 2b, and 21 (42.9%) patients were treated with pegylated (PEG)-IFN-α 2b. Five patients (10.2%) received natural (n) IFN-α, and others were treated with rIFN-α 2a (2.0%) and PEG-IFN-α 2a (2.0%), respectively. All interferons have nearly the same efficacy and bring about the same activation of the KYN pathway^52^. The regular duration of treatment was 48 weeks for PEG-IFN-α 2a and 2b at a dose of 180 μg (1.5 μg/kg)/week, and ribavirin at a dose of 600 mg/day (body weight ≤60 kg), or 800 mg/day (body weight 61–80 kg), or 1000 mg/day (body weight >80 kg). The duration of other IFN-α treatments, including rIFN-α 2a, 2b, and nIFN-α, was normally 24 weeks at a dose of 300–900 MIU/day, with or without ribavirin (800 mg/day). No patient had a past history of psychiatric treatment, and all were free from depression before IFN-α therapy. No patients received antidepressant medication during the study period. At an average of 104.2 ± 15.8 days after the onset of IFN-α therapy, some patients presented with depressed mood, apathy, melancholy, social isolation tendencies, and an intention to stop taking IFN. The patients who felt depressed mood were referred for psychiatric evaluation and diagnosed with MDD by a psychiatrist. Nineteen of the HCV patients were diagnosed with depressive symptoms [depression (+)] while 30 of them exhibited no depressive symptoms [depression (−)]. Based on clinical interviews, diagnosis to verify the incidence of depressive symptoms for MDD was made according to the DSM-IV (Diagnostic and Statistical Manual of Mental Disorders fourth edition) and ICD-10 (International Statistical Classification of Disease and Related Health problems-10). Informed consent was obtained from each patient. The study protocol conformed to the ethical guidelines of the 1975 Declaration of Helsinki as reflected in a priori approval by the medical research ethics committee of Kyoto University and Gifu Municipal Hospital, and all experiments were performed in accordance with approved guidelines and regulations.

#### Preparation of serum samples from HCV patients

For all HCV patients, blood was collected before the onset of IFN-α therapy and 2 and 4 weeks after the onset of the therapy, and there was a no significant time difference for sampling blood between depression (−) and (+) patients (see Supplemental Table 1S and Fig. 2S). Serum was separated from the blood by low-speed centrifugation (800 × *g*, 15 min) at 4 °C and stored at −80 °C until analysis. The frozen serum was thawed at room temperature and then mixed with one volume of 10% (v/v) perchloric acid for deproteinization. After incubation for 10 min on ice, the mixture was centrifuged at 14,000 × *g* for 10 min at 4 °C, and the resultant supernatant was used for the measurement of TRP and its metabolites KYN, KA, AA, 3-HK, and 3-HAA.

### Experimental animals

All mice were 8-week-old males. *IDO1* K.O. mice, of a C57BL/6J background were obtained from Jackson Laboratory (Bar Harbor, ME). Wild -type C57BL/6J mice were obtained from SHIMIZU Laboratory Supplies Co., Ltd. (Kyoto, Japan). Animals were housed in the Kyoto University School of Medicine animal facilities under specific pathogen-free conditions and were maintained on a 12-hour light/dark cycle (lights on at 8:00 a.m.) at 25 °C. Mice had free access to food and water. The protocols for animal experiments were approved by the Animal Experimentation Committee of the Graduate School of Medicine, Kyoto University, and all experiments were performed in accordance with approved guidelines and regulations.

### *In vivo IFN*-γ gene transfer

Plasmid pCpG-Muγ was constructed by inserting a BglII/NheI murine *IFN*-γ cDNA fragment into the BglII/NheI site of the pCpG-mcs vector (InvivoGen, San Diego, CA) as described previously^53^,^54^. The prepared plasmid pCpG-Muγ was dissolved in normal saline and injected into the tail veins of the mice over 5 sec on day 0^55^,^56^. The injection volume was approximately 9% (v/w) of body weight. The doses of pCpG-Muγ used were 0.01, 0.05, and 0.1 pmol/mouse. To eliminate the possibility of tissue damage and/or inflammation by the hydrodynamic injection, a control plasmid, which was the empty vector without the *IFN*-γ gene (pCpG-mcs), was injected (0.05 pmol/mouse) [IFN-γ transfected (−) mice]. We confirmed that the injected plasmid pCpG-Muγ [IFN-γ transfected (+) mice] significantly increased IDO1 activity in the lung and frontal cortex at 0.05 or 0.1 pmol/mouse (see Supplemental Fig. 5S) compared to IFN-γ transfected (−) mice. Therefore, the plasmid dose was fixed at 0.05 pmol/mouse throughout all experiments, which corresponded to 0.10–0.12 μg of DNA/mouse.

### Behavioral tests

All behavioral tests were performed between 9:00 a.m. and 6:00 p.m. 4 weeks after *IFN*-γ gene transfer. The behavioral tests were conducted in the following order: the Y-maze test, OFT, and then FST.

Spontaneous alternation behavior of mice in a Y-maze, an index of short-term memory, was performed according to the method outlined in previous reports^57^,^58^. The Y-maze apparatus consisted of black-painted plywood, with each arm measuring 40 × 10 × 12 cm (L × W × H), tapering to 3-cm wide at the bottom. The arms converged to a triangular center, 4 cm per side. Each mouse was placed at the end of an arm and allowed to move freely throughout the maze during an 8-min session. The series of arm entries was recorded visually. Spontaneous alternation behavior was defined as the entry into all three arms (i.e., arm A, arm B, and arm C) on consecutive choices in triplet sets (i.e., ABC, ACB, BAC, BCA, CAB and CBC). Alternation behavior was calculated as the ratio of actual alternations to possible alternations (defined as the total number of arm entries-2) ×100, and listed as a percentage, as described previously^57^,^58^.

The OFT using a Letica model LE 8811 (Bioseb, France) was performed according to the method described previously^59^. To measure locomotor activity in a novel environment, a mouse was placed in a transparent acrylic cage with a black frosted Plexiglas floor (45 × 45 × 30 cm), and locomotion behaviors and rearing were measured every 1 min for 10 min using digital counters with infrared sensors (SCANET MV-40 OF; MELQUEST Co., Ltd., Toyama, Japan).

The FST is a standardized test of depressive-like behavior for which depression is inferred from increased durations of immobility. This test was conducted as described previously^60^, with slight modifications. Each mouse was placed in a transparent glass cylinder (20 cm high, 8 cm in diameter) that contained water at 22 ± 1 °C to a depth of 13.5 cm and was forced to swim for 300 sec. The duration of swimming was measured using a SCANET MV-40 AQ apparatus (MELQUEST Co., Ltd., Toyama, Japan). The duration of immobility was recorded during the last 5 min of the 6-minute test. The mice were subjected to 15 min training under similar conditions, 24 h before the test.

### Preparation of serum and tissue samples from experimental animals

Immediately after the end of all behavioral tests, mice were sacrificed under sodium pentobarbital (50 mg/kg of body weight, i.p.) anesthesia, and blood was collected from the abdominal vena cava. Serum was separated from the blood by low-speed centrifugation (1,000 × *g*, 10 min). Serum samples for the measurements of TRP and its metabolites were prepared in the same way as for HCV patients.

Immediately after sacrifice, the whole brain was isolated from each mouse, and the frontal cortex was dissected from the brain. Each frontal cortex was placed in a polypropylene tube and immediately frozen by immersion in liquid nitrogen and kept at −80 °C until analysis. Tissue samples for measurements of TRP and its metabolites were prepared by homogenizing the frontal cortex in 1.5 volumes of 3% perchloric acid. Each homogenized sample was maintained at 4 °C overnight and then centrifuged at 12,000 × *g* for 20 min at 4 °C. The resultant supernatant was loaded onto an Ultrafree-MC Centrifugal Filter Unit with a Microporous Membrane (0.45 μm Cat UFC30HV00, Millipore) and centrifuged at 12,000 × *g* for 4 min at 4 °C.

### Laboratory assessments

All biological samples were analyzed by research staff blinded to the clinical and experimental status of study participants.

### Measurements of TRP and its metabolites

TRP, KYN, KA, AA, 3-HK, and 3-HAA in the serum of HCV patients and the serum and frontal cortex of mice were measured by high-performance liquid chromatography (HPLC) using a spectrophotometric (UV detector, SPD-20A, Shimadzu Co., Kyoto, Japan) or a fluorescence spectrometric detector (RF-10AXL, Shimadzu Co., Kyoto, Japan). TRP, KYN, AA, and KA were separated on a reverse phase chromatography column (TSK-GEL ODS-100 V 3 μm, 150 mm × 4.6 mm, i.d. 3 μm particle size, TOSOH Co., Tokyo, Japan) with a mobile phase of 100 mM zinc acetate, 10 mM sodium acetate (pH6.2), and 2% (v/v) acetonitrile at a flow rate of 1.0 ml/min. UV signals initially were monitored at 365 nm to detect KYN. After 18 min, UV was measured at 230 nm to detect TRP. The fluorescence excitation and emission wavelengths were set at 320 and 420 nm for 3-HAA and AA, respectively. After 15 min, excitation and extension wavelengths were changed to 344 and 404 nm, respectively, for KA. 3-HK was measured by HPLC with an electrochemical detector, Eicom ECD-300 (+550 mV) and a chromatographic column (EICOMPAK SC-50DS, 150 mm × 3.0 mm, i.d. 3 μm particle size, Eicom Co., Kyoto, Japan) with a mobile phase of 0.59% (v/v) phosphoric acid, 0.34 mM EDTA, 14 mM heptanesulfonic acid, 0.9% (v/v) triethtlamine and 1% (v/v) acetonitrile at a flow rate of 0.5 ml/min. In the clinical samples, some metabolites were difficult to separate clearly by HPLC, and we excluded the data which did not show the single peak.

### Measurements of 5-HT and 5-HIAA

5-HT and 5-HIAA were measured in the frontal cortex of mice by the method described previously^61^. Briefly, 5-HT and 5-HIAA were determined using HPLC with an electrochemical detector, Eicom ECD-300 (+750 mV), and a chromatographic column (EICOMPAK SC-50DS, 150 mm × 3.0 mm, i.d. 3 μm particle size, Eicom Co., Kyoto, Japan) with a mobile phase of 83% (v/v) 0.1 M acetic acid-citric acid buffer (pH3.5) containing 0.88 mM sodium 1-octanesulfonate, 0.015 mM EDTA, and 17% (v/v) methanol at a flow rate of 0.5 ml/min. Each frozen brain sample was weighed and homogenized with an ultrasonic processor in 0.2 M perchloric acid containing isoproterenol as an internal standard. The homogenates were placed on ice for 30 min and centrifuged at 20,000 × *g* for 15 min. The resultant supernatants were mixed with 1 M sodium acetate to adjust the pH to 3.0 and injected into an HPLC system. The serotonin turnover was estimated by the ratio of 5-HIAA/5-HT.

### Statistical analysis

The data (mean ± SEM) from patients were analyzed by group, controlling for age and gender. The comparison of the mean values of the parameters, which showed normal distributions between depression (+) and depression (−) patients, was performed using Student’s *t*-test, the Mann-Whitney test, and a one-way analysis of variance (ANOVA) with the Bonferroni post hoc test. A two-tailed *p* value of less than 0.05 was considered statistically significant. Results from animal experiments were also expressed as mean ± SEM. Intergroup comparisons were made using one-way or two-way ANOVAs, followed by the Bonferroni’s/Dunn’s multiple range test. Statistical analyses and graphing were performed using GraphPad Prism version 5.0 (GraphPad Software, San Diego, USA). A *p* value of less than 0.05 was considered statistically significant.

## Additional Information

**How to cite this article**: Murakami, Y. *et al*. Depressive symptoms as a side effect of Interferon-α therapy induced by induction of indoleamine 2,3-dioxygenase 1. *Sci. Rep.* **6**, 29920; doi: 10.1038/srep29920 (2016).

## Supplementary Material

Supplementary Information

## Figures and Tables

**Table 1:** Clinical characteristics of HCV patients undergoing IFN-α therapy.

	Depression (−)	Depression (+)
All subjects	30 (Male: 20; Female: 10)	19 (Male: 12; Female: 7)
Age	54.33 ± 2.06	54.0 ± 2.29
HCV genotype 1b	24 (80%)	15 (78.9%)
HCV genotype 2a	4 (13.3%)	3 (15.8%)
HCV genotype 2b	2 (6.7%)	1 (5.3%)
AST	59.43 ± 5.09	57.47 ± 6.45
ALT	82.68 ± 11.36	69.56 ± 8.65

“Depression (−)”: HCV patients without depression, “Depression (+)”: HCV patients with depression following IFN-α therapy.

HCV; hepatitis C virus, AST; aspartate aminotransferase, ALT; alanine aminotransferase.

**Table 2:** Changes in the levels of serum TRP and its metabolites in HCV patients undergoing IFN-α therapy.

	% of value before IFN-α therapy		t	df	*p* value
	Depression (−)	Depression (+)			
**2 w after onset of therapy**					
TRP	95.4 ± 2.93	100.5 ± 3.98	0.965	40	0.340
KYN	108.6 ± 4.77	118.1 ± 4.24^*^	1.200	39	0.237
3-HK	117.0 ± 7.13	152.6 ± 10.4^***, ##^	2.886	38	0.006
KA	97.4 ± 5.51	95.9 ± 7.13	0.136	38	0.892
AA	119.9 ± 7.42	115.5 ± 7.11	0.381	41	0.706
3-HAA	102.9 ± 6.53	121.8 ± 12.6	1.452	37	0.155
**4 w after onset of therapy**					
TRP	92.0 ± 2.55	93.3 ± 6.49	0.213	39	0.833
KYN	104.8 ± 4.38	114.4 ± 6.38	1.204	39	0.236
3-HK	123.0 ± 9.01	155.0 ± 11.5^***^	2.005	36	0.053
KA	91.9 ± 5.12	88.8 ± 6.98	0.341	40	0.735
AA	107.5 ± 5.32	103.6 ± 11.3	0.361	40	0.720
3-HAA	101.9 ± 6.52	104.5 ± 14.8	0.182	36	0.857

Percent value of serum TRP, KYN, 3-HK, KA, AA, and 3-HAA concentrations in HCV patients at 2 and 4 weeks after onset of therapy, compared with the concentration (100%) before IFN-α therapy. In the clinical samples, some metabolites were difficult to separate clearly by HPLC. Therefore, the degree of freedom (df) values differ by the measured molecules. “Depression (−)”: the HCV patients without depression, “Depression (+)”: the HCV patients with depression. ^*^p < 0.05, ^***^p < 0.001 *versus* before the therapy, ^##^*p* < 0.01 *versus* Depression (−).

**Table 3:** Changes in serum KYN/TRP and 3-HK/KA ratios in HCV patients undergoing IFN-α therapy.

	% of value before IFN-α therapy		t	df	*p* value
	Depression (−)	Depression (+)			
**2 w after onset of therapy**					
KYN/TRP	115.6 ± 4.55	114.1 ± 5.95	0.198	42	0.844
3-HK/KA	129.1 ± 9.52	144.0 ± 9.06^*^	1.036	39	0.308
**4 w after onset of therapy**					
KYN/TRP	115.7 ± 5.69	138.3 ± 8.84^*,#^	2.094	35	0.044
3-HK/KA	129.6 ± 8.67	171.1 ± 18.6^***,#^	2.325	35	0.026

Serum KYN/TRP, reflecting IDO1 activity, and 3-HK/KA, reflecting neurotoxic indices, both ratios in HCV patients were shown as % of value compared with the value (100%) before IFN-α therapy, at 2 and 4 weeks after onset of therapy. ^*^p < 0.05, ^***^p < 0.001 *versus* before the therapy, ^#^*p* < 0.05 *versus* Depression (−).
