# Supplementary material for: Depressive symptoms as a side effect of Interferon-α therapy induced by induction of indoleamine 2,3-dioxygenase 1
Source: Sci Rep. 2016 Jul 20;6:29920. doi: 10.1038/srep29920 (PMC4951771; doi:10.1038/srep29920)
Supplement: Supplementary Information [file srep29920-s1.pdf]

Supplementary information

The manuscript title: Depressive symptoms as a side effect of Interferon-α therapy induced by induction of indoleamine 2,3-dioxygenase 1

Author list: Yuki Murakami[1, 2 *], Takaaki Ishibashi[1, *], Eiichi Tomita[3], Yukio Imamura[1, 4], Tomoyuki Tashiro[1], Kanitta Watcharanurak[5], Makiya Nishikawa[5], Yuki Takahashi[5], Yoshinobu Takakura[5], Satoko Mitani[1, 6], Hidetsugu Fujigaki[7],Yoshiji Ohta[8], Hisako Kubo[1], Takayoshi Mamiya[9, 10], Toshitaka Nabeshima[10, 11,12], Hyoung-Chun Kim[13], Yasuko Yamamoto[1] and Kuniaki Saito[1, 8, 10]

[1] Human Health Sciences, Graduate School of Medicine and Faculty of Medicine, Kyoto University, Kyoto 606-8507, Japan.

[2] Organization for Research Initiatives and Development, Doshisha University, Kyoto 610-0394, Japan.

[3] Department of Gastroenterology, Gifu Municipal Hospital, Gifu 500-8513, Japan.

[4] Labratory of Nano-bio Probe, Quantitative Biology Center (QBic), RIKEN, Osaka 565-0874, Japan.

[5] Department of Biopharmaceutics and Drug Metabolism, Graduate School of Pharmaceutical Sciences, Kyoto University, Kyoto 606-8501, Japan.

[6] Department of Nursing School of Health Sciences, Gifu University of Medical Science, Gifu 501-3892, Japan.

[7] Department of Medical Science and Technology, Faculty of Health Sciences, Hiroshima International University, Hiroshima, 739-2695, Japan.

[8] Department of Medical Sciences Innovation, Fujita Health University Graduate School of Health Sciences, Aichi 470-1192, Japan.

[9] Department of Chemical Pharmacology, Faculty of Pharmacy, Meijyo University, Aichi 468-8503, Japan.

[10] Japanese Drug Organization of Appropriate Use and Research, Aichi 468-0069, Japan.

[11] Advanced Diagnostic System Research Laboratory, Fujita Health University Graduate School of Health Sciences, Aichi 470-1192, Japan.

[12] Aino University, Osaka 567-0012, Japan.

[13] Neuropsychopharmacology & Toxicology Program, College of Pharmacy, Kangwon National University, Gangwon 200-701, Republic of Korea.

Short title: Role of IDO1 in IFN-α-Induced Depressive Symptoms (49 characters)

* These authors contributed equally to this work.

Correspondence to Yuki Murakami, Ph.D: ymurakam@mail.doshisha.ac.jp,

Organization for Research Initiatives and Development, Doshisha University, Kyoto,

Japan. Phone: +81-774-65-6060, Fax: +81-774-73-1903.

Supplemental Methods

Comparison between the effect of IFN-α and IFN-γ administration on the induction of IDO1 activity in mice

Mouse recombinant (mr) IFN-α was acquired from Pestka Biomedical Laboratories, Inc. (NJ, USA) and mrIFN-γ was obtained from Wako Pure Chemical Industries, Ltd. (Osaka, Japan). Both rIFNs were dissolved in PBS(-). Mice were given a single intraperitoneal (i.p.) injection of either mrIFN-α (5,000 IU/mouse), mrIFN-γ (5,000 IU/mouse) or PBS (-) as a control. Blood samples were collected 24 h after i.p. injection. All mice were sacrificed under sodium pentobarbital (50 mg/kg of body weight, i.p.) anesthesia, and blood was collected from the abdominal vena cava. Serum was separated from the blood by low-speed centrifugation (1,000 x *g* for 10 min). Serum samples for the measurements of TRP and KYN were prepared in the same way as HCV patients.

Dose dependency and chronic administration effects of IFN-γ on IDO1 activity

To examine the effect of IFN-γ dose dependency on IDO1 activity, mice were given a single i.p. injection of different dose of mrIFN-γ (1,250, 2,500, 5,000, and 10,000 IU/mouse) or PBS (-) as a control. Furthermore, to evaluate the chronic administration effect of IFN-γ on IDO1 activity, mice were given i.p. injections of either mrIFN-γ (5,000 or 10,000 IU/mouse) or PBS (-) once every 3 days for 9 days (total of 4 injections). Blood and tissue samples were collected 24 h after the last injection. All mice were sacrificed under sodium pentobarbital (50 mg/kg of body weight, i.p.) anesthesia and blood was collected from the abdominal vena cava. Serum was separated from the blood by low-speed centrifugation (1,000 x *g* for 10 min). Serum samples for the measurements of TRP and KYN were prepared in the same way as HCV patients. Tissue samples for measurement of IDO1 activity were immediately frozen by immersion in liquid nitrogen and kept at -80˚C until analysis.

Determination of IDO1 enzyme activity in the lung and brain after the administration of mrIFN-γ or IFN-γ gene transfer

IDO1 activity was measured as described previously[1]. Briefly, tissues were disrupted

with a ploytron homogenizer in 2-5 volumes of ice-cold 0.14 M KCl/20 mM potassium phosphate buffer (pH7.0). The homogenates were centrifuged at 20,000 x *g* for 10 min at 4˚C. Samples of supernatant were taken for measurement of IDO1 activity. The reaction mixture consisted of 50 μL enzyme preparation and 50 μL substrate solution. The composition of reaction mixture was 100 mM potassium phosphate buffer (pH 6.5), 50 mM methylene blue, 20 mg of catalase, 50 mM ascorbate and 0.4 mM L-TRP. The reaction mixture was incubated at 37˚C for 0 and 30 min. After incubation, samples were acidified by the addition of 100 μL of 3% perchloric acid and centrifuged at 20,000 x *g* for 10 min at 4˚C. The enzymatic products were measured by HPLC. Enzyme activity was determined by calculating the difference between product levels after 0 min and 30 min incubation and expressed as amount formed per hour per milligram of protein.

Supplemental References

1 Fujigaki, S. *et al.* Lipopolysaccharide induction of indoleamine 2,3-dioxygenase is mediated dominantly by an IFN-gamma-independent mechanism. *Eur J Immunol* **31**, 2313-2318, doi:10.1002/1521-4141(200108)31:8<2313::AID-IMMU2313>3.0.CO

;2-S (2001).

2 Saito, K., Markey, S. P. & Heyes, M. P. Chronic effects of gamma-interferon on quinolinic acid and indoleamine-2,3-dioxygenase in brain of C57BL6 mice. *Brain Res* **546**, 151-154 (1991).

Supplemental Figure Legends

Fig. 1S. Schematic overview of the KYN pathway. IDO1 catabolizes L-TRP to N-formyl-L-kynurenine, which is converted to L-KYN by formamidase. L-KYN is further metabolized to AA by kynureninase, to KA by Kynurenine aminotransferases (KATs), and to 3-HK by kynurenine 3-monooxygenase (KMO), which is metabolized to 3-HAA by 3-hydroxyanhranilate 3,4-dioxygnase. 3-HAA is further metabolized to QUIN.

Fig. 2S. Schematic depiction of the collection schedule for blood sampling from depression (-) and depression (+) HCV patients. The range of time points and average collection time point (a-d) per group are listed in Supplemental Table 1S.

Fig. 3S. Significant increases in IDO1 activity in mice following intraperitoneal administration of IFN-γ. We compared the induction of IDO1 activity in mice by a single i.p. injection of mrIFN-α or mrIFN-γ. We determined TRP and KYN concentrations and the KYN/TRP ratio, reflecting IDO1 activity in serum, 24 h after the administration of mrIFN-α, or -γ. rmIFN-γ significantly increased the activity of IDO1, whereas mrIFN-α showed a weak direct IDO1 induction in mice. Each column represents the mean ± SEM (n=4-6). $^{***}p<0.001$ *versus* PBS (-) injected mice, $^{\#\#}p<0.01$, $^{\#\#\#}p<0.001$ *versus* mrIFN-α injected mice.

Fig. 4S. Increase of IDO1 activity in a dose-dependent manner after single and repeated-injections of mrIFN-γ in mice. (a, b) To examine the effect of IFN-γ dose dependency on IDO1 activity, mice were given a single i.p. injection of different doses of mrIFN-γ (1,250, 2,500, 5,000, and 10,000 IU/mouse) or PBS (-) as a control. (c, d) To evaluate the chronic administration effect of IFN-γ on IDO1 activity, mice were given i.p. injections of either mrIFN-γ (5,000 or 10,000 IU/mouse) or PBS (-) once every 3 days over a 10-day period (total of 4 injections). We determined TRP and KYN concentrations and KYN/TRP ratio, reflecting IDO1 activity in serum (a, c) and IDO1 activity in the lung and cortex (b, d). mrIFN-γ increased IDO1 activity in a

dose-dependent manner in mice. Repeated administration of mrIFN-γ to mice increased IDO1 activity in both lung and cortex, as previously shown[2]. Each column represents the mean ± SEM (n=3-5). *p<0.05, **p<0.01, ***p<0.001 *versus* PBS (-) injected mice, $^{\#}p$<0.05, $^{\#\#\#}p$<0.001 *versus* 5,000 IU mrIFN-γ injected mice.

Fig. 5S. Increase of IDO1 activity in the lung and cortex of mice after *IFN-γ* gene transfer. To determine the optimum concentration of pCpG-Muγ plasmid to use, we evaluated (a) TRP and KYN concentrations and KYN/TRP ratio, reflecting IDO1 activity, in serum and (b) the IDO1 activity in the lung and cortex after hydrodynamic injection of three different plasmid doses. IFN-γ-transfected (-) mice were injected with the control plasmid pCpG-mcs, which is an empty vector, and IFN-γ-transfected (+) mice were injected with the pCpG-Muγ plasmid, which continuously expressed murine IFN-γ. The Y axis shows IDO1 enzyme activity (nmol/h/mg protein). IDO1 activity was increased in the lung and cortex of mice after *IFN-γ* gene transfer in a dose-dependent manner. Each column represents the mean ± SEM (n=5-6). *p<0.05, **p<0.01, ***p<0.001 *versus* IFN-γ-transfected (-) mice.

Supplemental Table

Table 1S Time points of clinical sample collections.

Time points	Depression (-) (mean ± SEM)	Depression (+) (mean ± SEM)	t	df	p value
(a) Before the onset of therapy	1-35 d (6.3 ± 1.8 d)	1-22 d (7.3 ± 1.4 d)	0.390	46	0.698
(b) 2 w after the onset of therapy	13-15 d (13.8 ± 0.1 d)	12-15 d (13.7 ± 0.2 d)	0.661	42	0.513
(c) 4 w after the onset of therapy	25-30 d (27.9 ± 0.1 d)	25 - 29 d (27.8 ± 0.3 d)	0.114	40	0.910
(d) The period of therapy	167-343 d (252.0 ± 15.7 d)	54 - 337 d (183.4 ± 22.0 * d)	2.592	46	0.013

For all HCV patients, blood was collected before the onset of IFN-α therapy, 2 and 4 weeks after the onset of therapy. Five patients with depression discontinued their treatment after the diagnosis of depressive symptom, therefore, the period of therapy was significantly different between the depression (+) and (-) groups. For the schedule of blood sampling, see Supplemental Fig. 2S online. *p<0.05 versus depression (-).

Fig. 1S

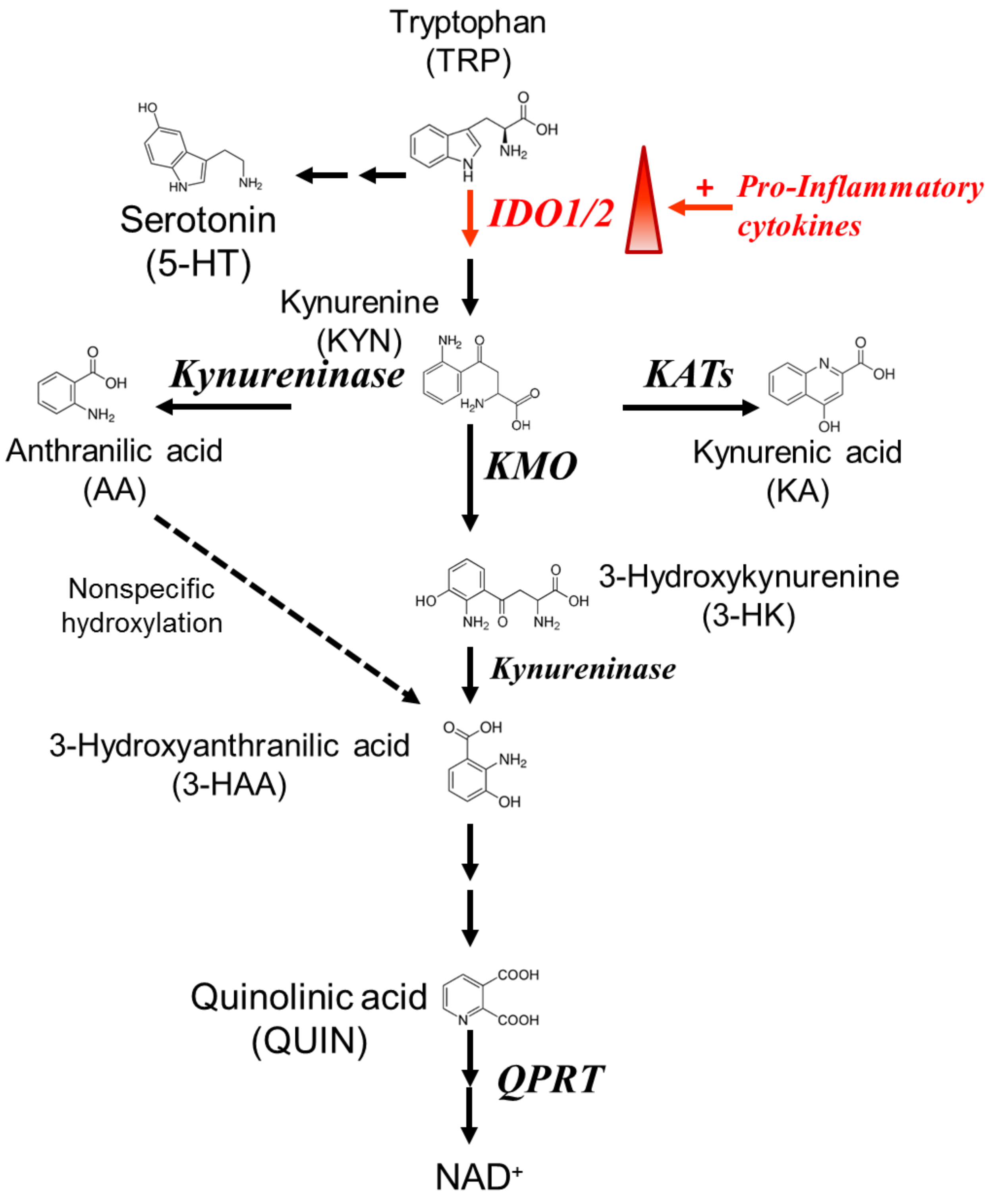

Tryptophan
(TRP)
Serotonin
(5-HT)
IDO1/2
+ Pro-Inflammatory
cytokines
Kynurenine
(KYN)
Kynureninase
KATs
Anthranilic acid
(AA)
KMO
Kynurenic acid
(KA)
3-Hydroxykynurenine
(3-HK)
Nonspecific
hydroxylation
Kynureninase
3-Hydroxyanthranilic acid
(3-HAA)
Quinolinic acid
(QUIN)
QPRT
NAD+

Fig. 2S

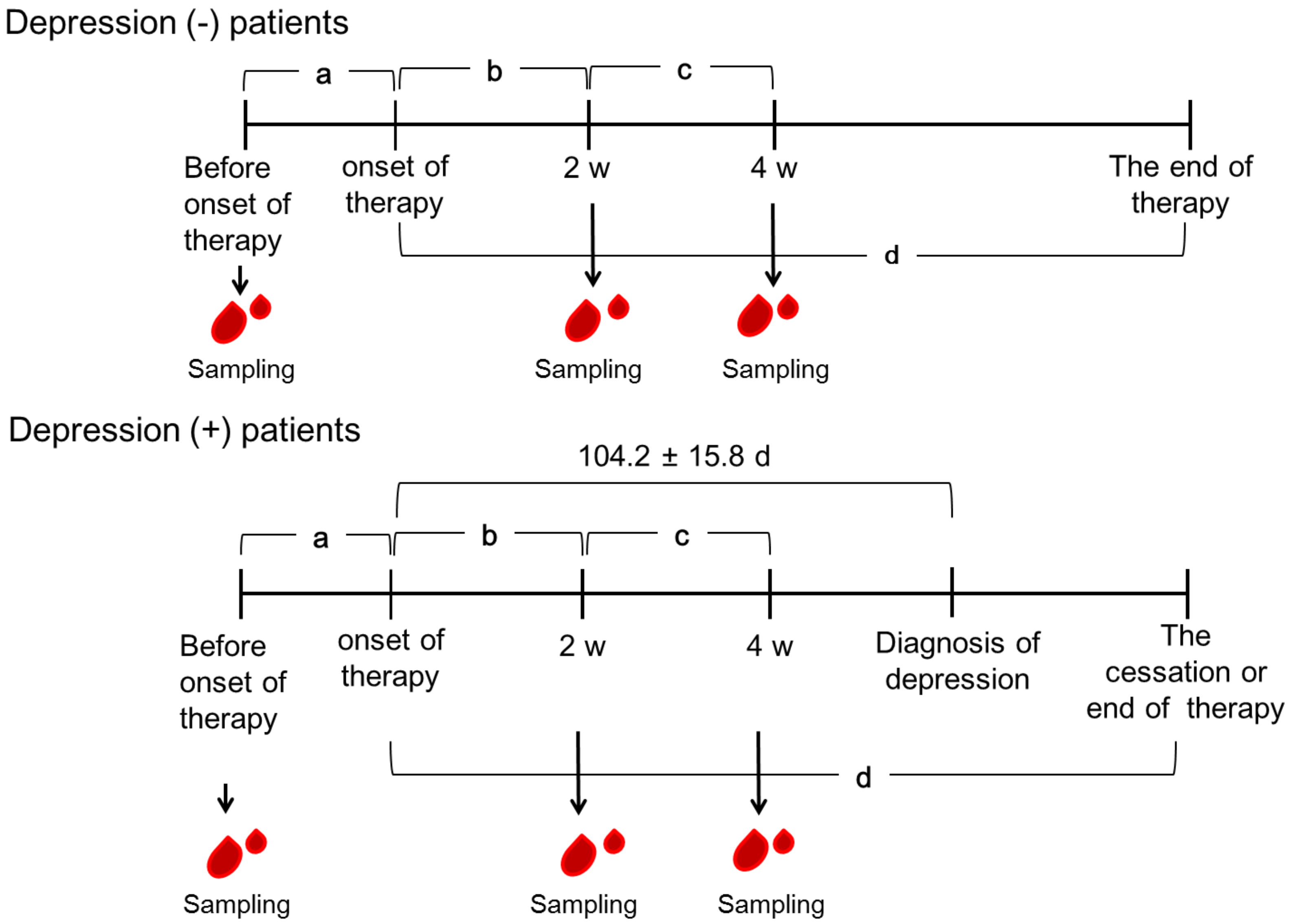
Depression (-) patients
a
b
c
Before onset of therapy
onset of therapy
2 w
4 w
The end of therapy
d
Sampling
Sampling
Sampling
Depression (+) patients
104.2 ± 15.8 d
a
b
c
Before onset of therapy
onset of therapy
2 w
4 w
Diagnosis of depression
The cessation or end of therapy
d
Sampling
Sampling
Sampling

Fig. 3S

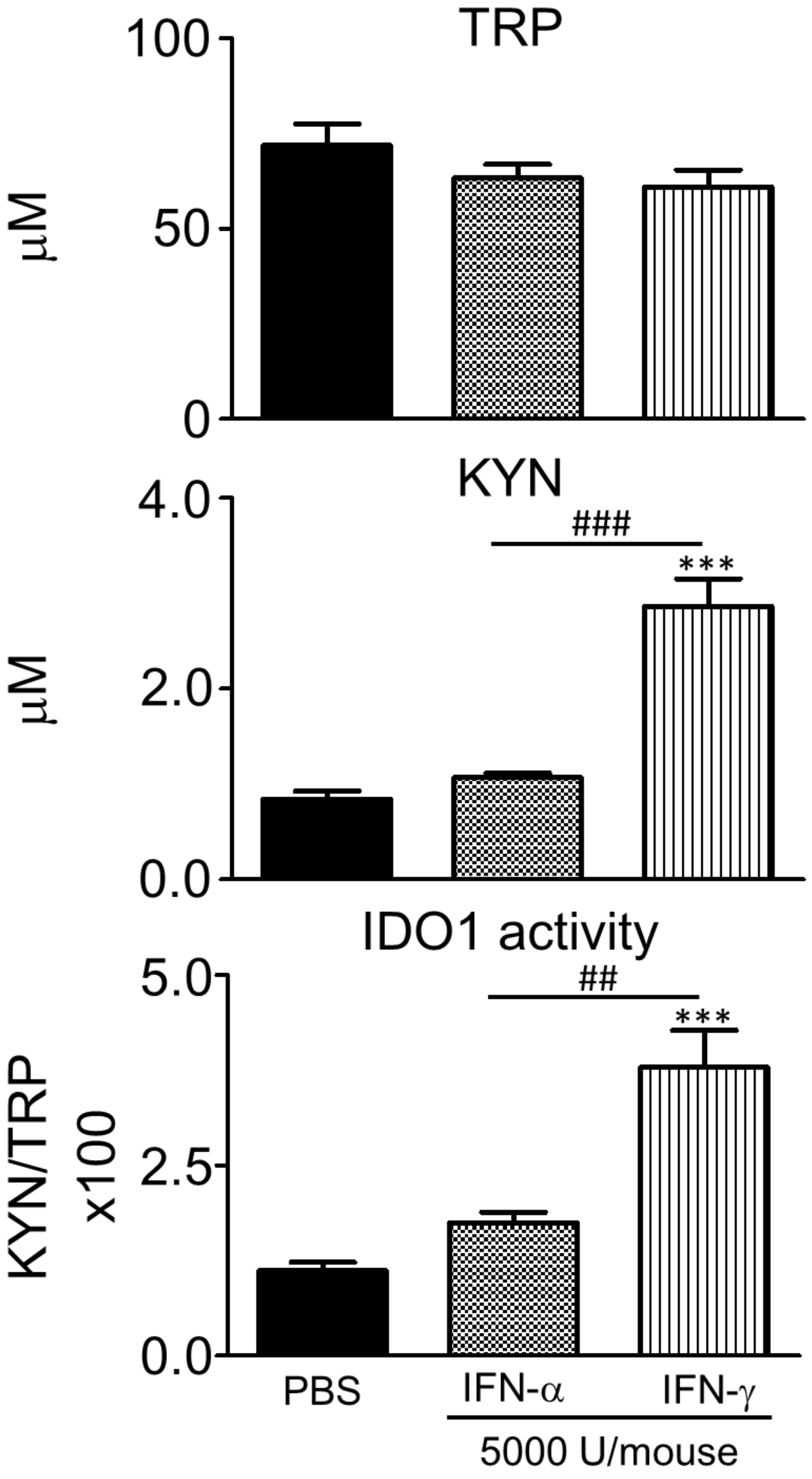
TRP
100
50
0
μM
KYN
4.0
2.0
0.0
μM
###
***
IDO1 activity
5.0
2.5
0.0
KYN/TRP
x100
##
***
PBS
IFN-α
IFN-γ
5000 U/mouse

Fig. 4S

(a) Serum

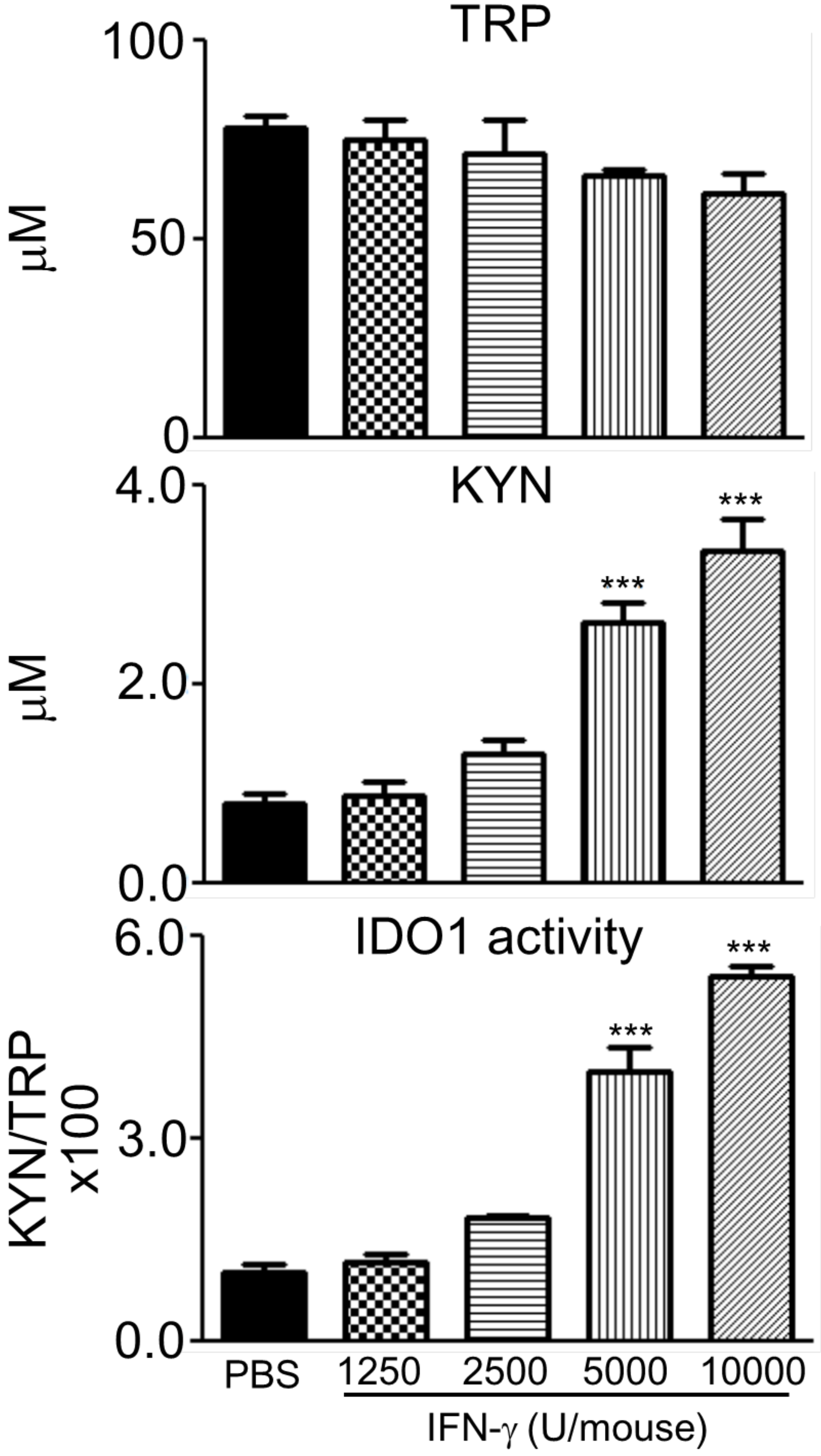

Fig. 4S

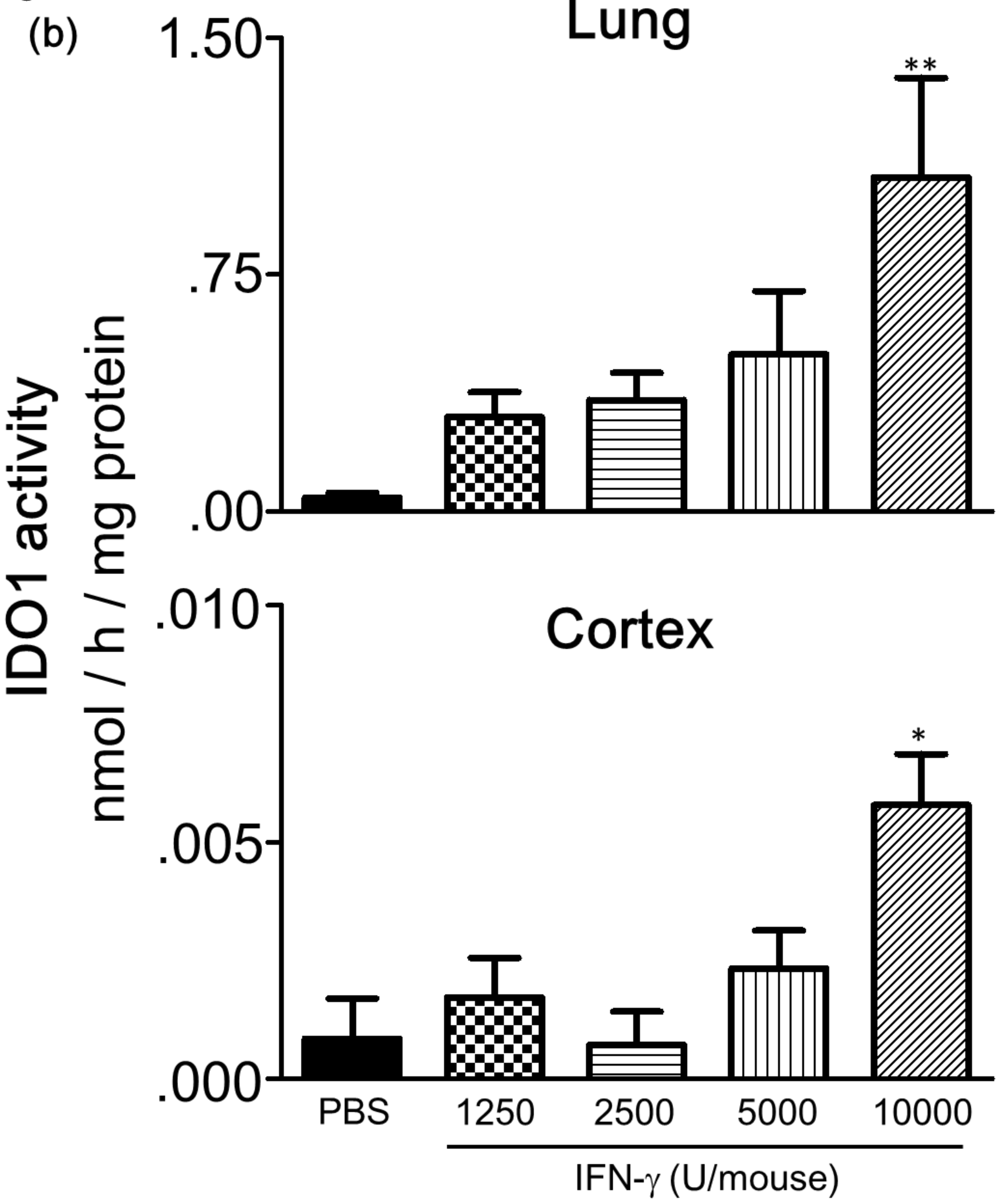
(b)
Lung
Cortex
IDO1 activity
nmol / h / mg protein
1.50
.75
.00
.010
.005
.000
**
*
PBS
1250
2500
5000
10000
IFN-γ (U/mouse)

Fig. 4S

(c) Serum

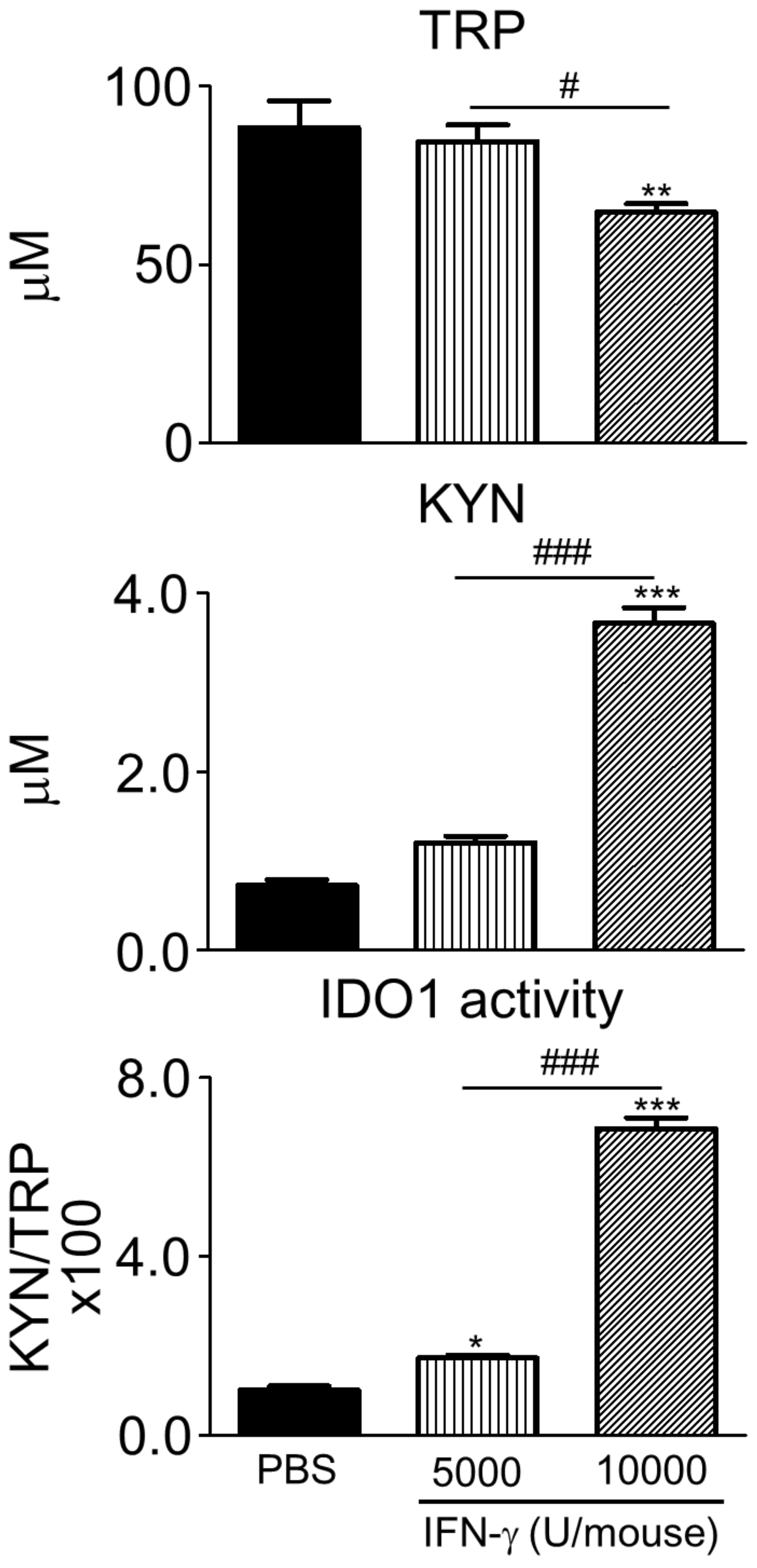

Fig. 4S

(d)

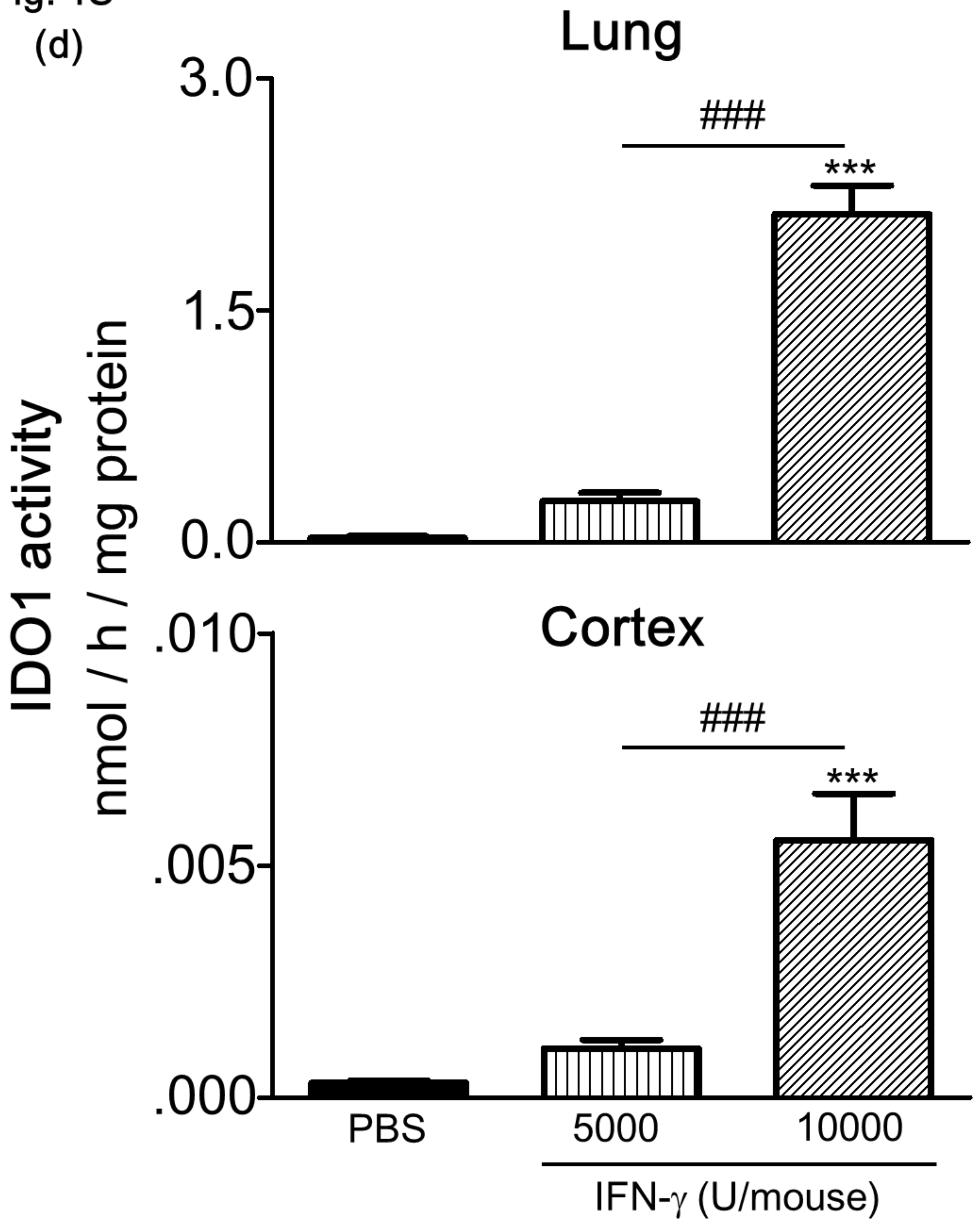
Lung
3.0
1.5
0.0
###
***
Cortex
.010
.005
.000
###
***
IDO1 activity
nmol / h / mg protein
PBS
5000
10000
IFN-γ (U/mouse)

Fig. 5S

(a) Serum

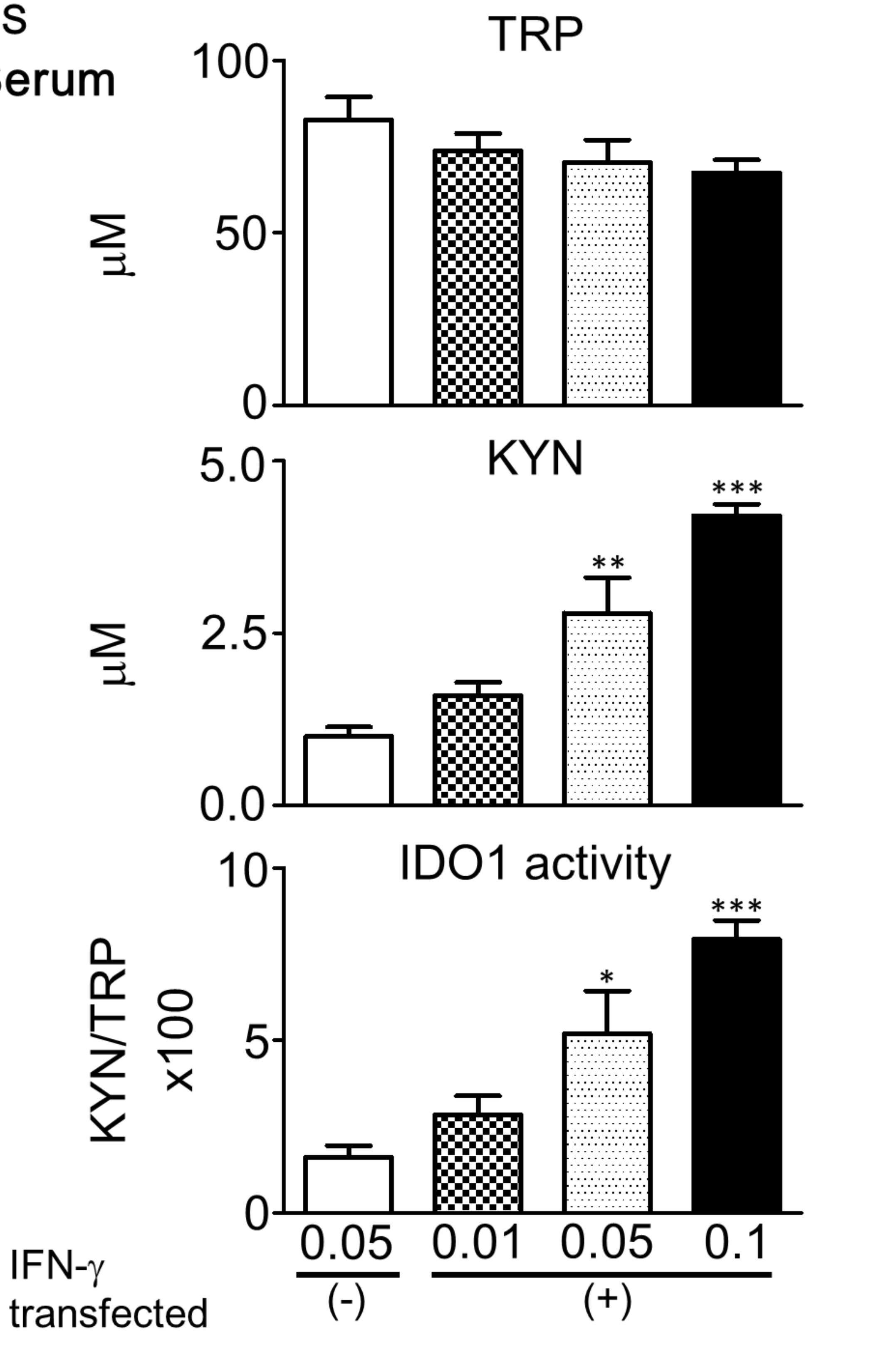

Fig. 5S

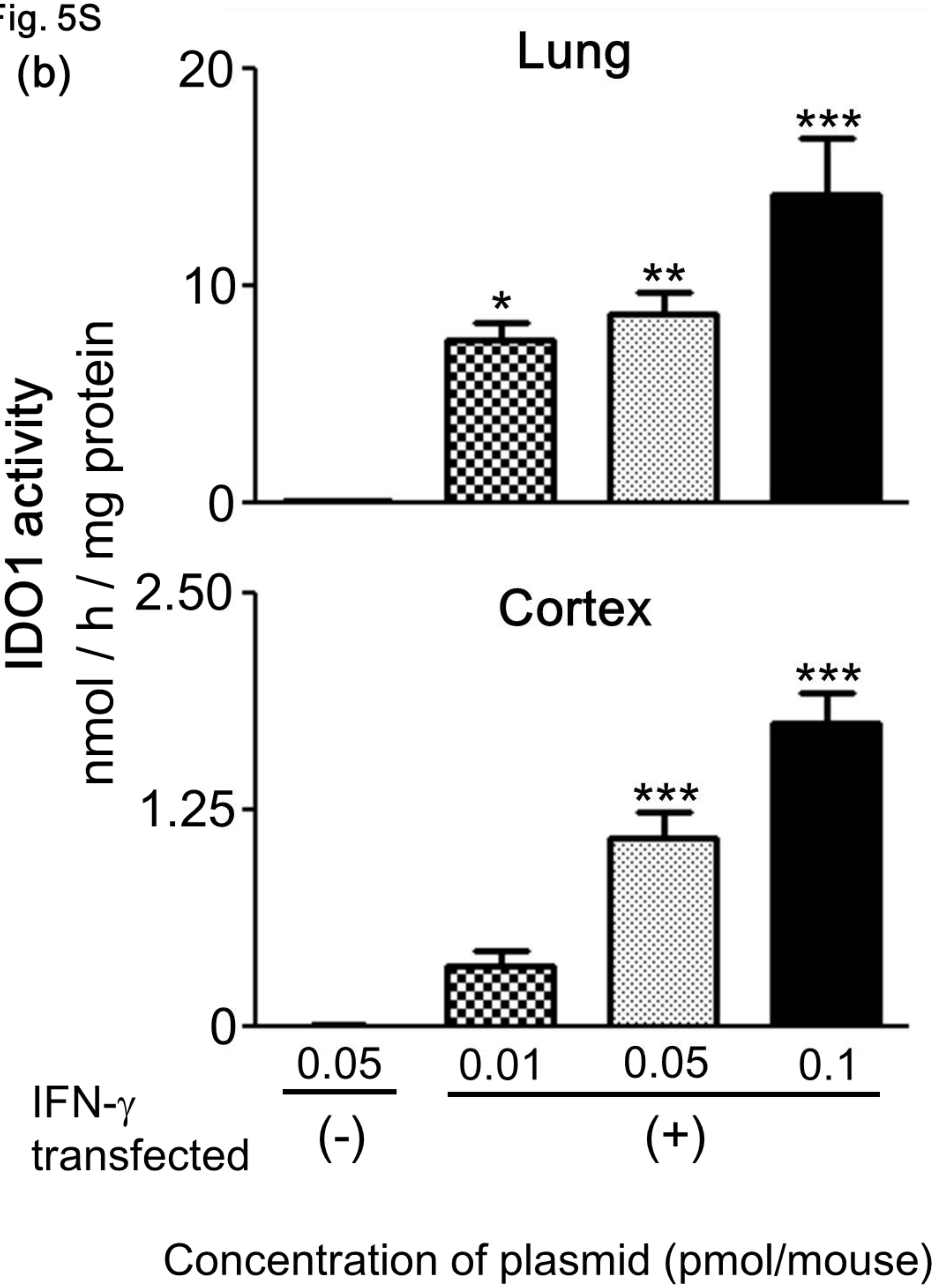
(b)
Lung
20
10
0
*
**
***
Cortex
2.50
1.25
0
***
***
IDO1 activity
nmol / h / mg protein
0.05
0.01
0.05
0.1
IFN-γ
transfected
(-)
(+)
Concentration of plasmid (pmol/mouse)
